# Geospatial analysis of the patterns of chemical exposures among biota in the Canadian Oil Sands Region

**DOI:** 10.1371/journal.pone.0239086

**Published:** 2020-09-30

**Authors:** Kristin M. Eccles, Bruce Pauli, Hing Man Chan

**Affiliations:** 1 Department of Biology, University of Ottawa, Ottawa, ON, Canada; 2 Science and Technology Branch, Environment and Climate Change Canada, National Wildlife Research Center, Ottawa, ON, Canada; Trent University, CANADA

## Abstract

Understanding the patterns of chemical exposure among biota across a landscape is challenging due to the spatial heterogeneity and complexity of the sources, pathways, and fate of the different chemicals. While spatially-driven relationships between contaminant sources and biota body burdens of a single chemical are commonly modelled, there has been little effort on modelling chemical mixtures across multiple wildlife species in the Canadian Oil Sands region. In this study, we used spatial principal components analysis (sPCA) to assess spatial patterns of the body burdens of 22 metals and Potentially Toxic Elements (PTEs) in 492 individual wildlife, including fur-bearing mammals, colonial waterbirds, and amphibians collected from the Canadian Oil Sands region in Canada. Spatial analysis and mapping both indicate that some of the complex exposures in the studied biota are distributed randomly across a landscape, which suggests background or non-point source exposures. In contrast, the pattern of exposure for seven metals and PTEs, including mercury, vanadium, lead, rubidium, lithium, strontium, and barium, exhibited a clustered pattern to the east of the open-pit mining area and in regions downstream of oil sands development which indicates point-source input. This analysis demonstrated useful methods for integrating monitoring datasets and identifying sources and potential drivers of exposure to chemical mixtures in biota across a landscape. These results can be used to support an adaptive monitoring program by identifying regions needing additional monitoring, health impact assessments, and possible intervention strategies.

## Introduction

Traditional risk assessments in ecotoxicology have focused on a chemical by chemical exposure approach [1]. While these simple models are easy to develop and interpret, they are not representative of the true nature of environmental chemical exposure in biota. As the biota in an ecosystem is often, if not always, exposed to environmental contaminant mixtures, better exposure models related to complex chemical mixtures are needed to reflect this ecological realism for valid risk assessments [2]. Complex exposures are particularly true for biota living in regions of high anthropogenic disturbance such as the Canadian Oil Sands, which is the third-largest reserve of crude oil in the world (after Venezuela and Saudi Arabia) [3]. The Canadian Oil sands, which is comprised of the Athabasca, Cold Lake, and Peace River deposits, covers approximately 142,200 km^2^ and is situated in the middle of the Canadian boreal forest [3]. The Athabasca Oil Sands Region (AOSR) is the most heavily disturbed, where approximately 4,800 km^2^ are undergoing surface mineable oil development, of which approximately 895 km^2^ have been cleared or disturbed [3, 4]. As a result, biota living in this region are exposed to many chemicals, including metals, primarily released during the upgrading of bitumen, and from fugitive dust related to mining activities [5–7].

The Canadian federal government and the Provincial Alberta Government initiated the Canada-Alberta Joint Oil Sands Monitoring (JOSM) program in 2012 to monitor the potential impact of oil sands development on the environment, including the potential impacts of contaminants of concerns associated with oil sands industrial operations on the health of wildlife in northern Alberta. JOSM included a total of five monitoring programs that examined the burdens of oil sands contaminants of concern in wildlife species. Annual sampling campaigns in the wildlife contaminants and toxicology monitoring program in JOSM measured a selected suite of chemical contaminants in wood frogs (*Lithobates sylvaticus*); fur-bearing mammals including river otter (*Lontra canadensis*), mink (*Neovision vision*), fisher (*Martes pennant*), and marten (*Martes Americana*); birds including the eggs of Caspian Terns (*Hydroprogne caspia*) and California Gulls (*Larus californicus*), and tree swallow (*Tachycineta bicolor*) nestlings; and a variety of aboveground and belowground plant matter. In the wildlife contaminants monitoring program, exposure to contaminants was measured in collected tissues, including liver, feathers, and eggs [8–13]. Programs under JOSM also monitored abiotic matrices, including air, water, and aerial deposition of contaminants, including in snow. The goal of these contaminant monitoring programs under JOSM was to improve the understanding of the potential impacts of chemicals released by the industry on the health of the ecosystems in the Oil Sands Region [14].

Environmental effects monitoring programs typically select bioindicator (also referred to as a sentinel) species and biomarkers based on their ability to provide information useful to make inferences about environmental conditions. These bioindicators and biomarkers are also used to make inferences about the health of ecosystems being monitored and humans living in who use those ecosystems [15, 16]. However, it is well established that a single species or biomarker cannot be representative of the health of the entire ecosystem. Therefore, data are collected from multiple biomarkers in multiple species across a landscape to gain a better understanding of the impacts contaminants of concern have on ecosystem health [17, 18]. However, the contaminant data collected by the various contaminants monitoring projects under JOSM have yet to be integrated and spatially analyzed [14].

Metals and Potentially Toxic Elements (PTEs), including arsenic, cadmium, cobalt, chromium, nickel, mercury, lead, and zinc, are naturally present in all ecosystems [19]. In some regions, such as the Canadian Oil Sands, metals and PTEs are being anthropogenically enriched and potentially increasing wildlife exposures [6, 20, 21]. While some metals (i.e., iron, copper, zinc) are essential micro-nutrients and are required at low doses to maintain physiological homeostasis, high exposures to these elements can be potentially toxic [22]. Further, non-essential metals (i.e., mercury, cadmium, lead) are not required for homeostasis and can be toxic even at low doses [22]. Two previous studies have assessed complex metal exposures in benthic organisms and small mammals using principal components analysis [23, 24]. However, wildlife contaminants monitoring data collected from the Canadian Oil Sands have yet to be integrated for exposure assessment.

The JOSM monitoring activities have created geo-referenced datasets of metal and PTE contaminant burdens and effects in the sentinel species. Therefore, questions related to the spatial distribution of contaminants in the environment or the relationships between contaminant exposure and effects in biota can be investigated using a common geospatial platform [25]. However, there are many analytical challenges to analyze the highly dimensional spatial environmental monitoring datasets. For example, different species and life stages will accumulate and detoxify contaminants at different rates and use different mechanistic pathways [26]. Further, chemicals are measured in different matrices (e.g., feather, muscle, liver). The species, life stage, and matrix differences in chemical concentrations can make it challenging to make direct comparisons between measured biomarkers and can hinder data integration [27]. Additionally, contaminant variables are often highly correlated, which can be problematic for some statistical methods such as regression [28].

Geographic information systems (GIS) are useful platforms to integrate data using a normalization method and assess spatial patterns of exposures to complex mixtures using a spatial principal component analysis (sPCA) [27]. The objective of this study is to demonstrate the use of a geospatial framework and spatial analytical methods to address the challenges associated with integrating and analyzing large wildlife datasets in ecotoxicology. Further, we also identify critical hotspots of metal contamination in wildlife collected as a part of the JOSM program in the Canadian Oil Sands and suggest possible drivers of the observed patterns.

## Materials and methods

### Data

Data were obtained from three wildlife contaminants monitoring projects (mammals, colonial waterbirds, and amphibians) that collected samples from these species under the JOSM wildlife contaminants monitoring program. The samples used in this study were collected between 2011 and 2016. The tadpole, recent metamorph, and adult wood frogs were ethically captured under permit in wetlands at varying distances from the surface mineable area. Whole-body samples were used for chemical analysis [9]. Wetlands are a receiving environment of meltwater of the winter snowpack and contaminants that were atmospherically deposited onto the snowpack [9]. The terrestrial and semi-aquatic fur-bearing mammals, including river otter, marten, mink, muskrat, and fisher, were trapped under permit for the commercial fur trade by Northern Alberta commercial trappers. All trapped mammals used in this study are adults. Carcasses were stored at − 20°C until the completion of gross necropsies, where the livers were dissected for chemical analysis [13, 29]. Fisher and marten favor old-growth coniferous forest with dense canopy cover [13] while the semi-aquatic furbearers inhabit the shorelines of rivers and lakes [30]. The eggs of gull and tern species were ethically collected under permit from nesting colonies. These colonial waterbirds nest along the shores and on islands of lakes and rivers in the region [10, 11, 21].

### Metal and PTE analysis

Briefly, the metal and PTE analyses of the sampled tissues were conducted at the National Wildlife Research Centre (Ottawa, ON, Canada). The laboratory is a CALA-accredited and ISO 17025 certified facility using a standardized operating protocol (SOP# MTH-MET-TE-01C0). Samples were acid-digested and analyzed using a PerkinElmer NexION 300d inductively-coupled plasma-mass spectrometer (ICP-MS) using the principles of the USEPA 6020 and 200.8 methods, with modifications for wildlife tissue. Quality assurance and control methods included the use of certified reference material (NIST CRM Oyster Tissue 1566b and CRM Lobster Hepatopancreas TorT-2) and an internal standard (Ge, Lu, Rh, and Sc mixed inhouse purchased from Delta Scientific, Mississauga, ON, Canada). The data were corrected for the percent recovery of each internal standard. Blanks (1% HNO^3^) were run at the beginning and the end of each set of nine samples, as well as before and after the standard calibration. Each sample was run in duplicate or triplicate. Metal and PTE results were expressed in μg/g dry weight (d.w.).

### Data pre-processing

**Fig 1:**
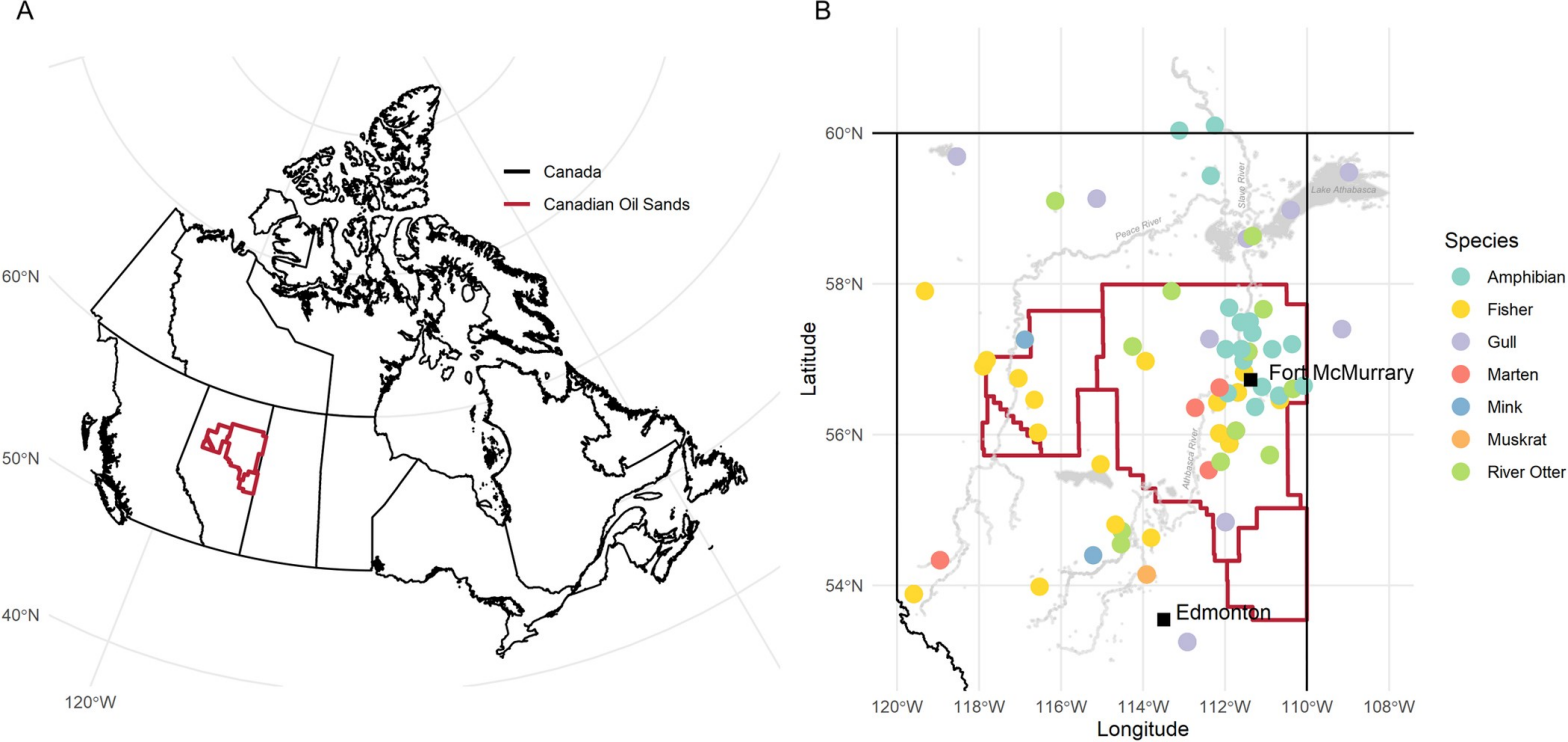
Overview of study location and samples. (A) The location of the Oil Sands Region within Canada. (B) Locations of the different collection locations of the species across the landscape (after data cleaning, n = 492). The figure is generated using the Statistics Canada GIS data (https://www12.statcan.gc.ca/census-recensement/2011/geo/bound-limit/bound-limit-2016-eng.cfm).

Once compiled, the dataset was cleaned prior to statistical analysis. Not all chemicals were analyzed in all samples. Thus, samples with missing data were removed. All measurements that were below the method detection limit (MDL) were recoded to half of the detection limit of each metal. All metals and PTEs that had a high proportion of measurements below the MDL (BDL) were removed from the dataset; beryllium (81% BDL), antimony (91% BDL), and uranium (96% BDL) were removed before analysis. In the cleaned dataset, there were 22 metal covariates with a sample size of 492. A summary of the species, life stages, and the number of samples from each monitoring project before data cleaning and after data cleaning is presented in Table 1. An overview of the study area, the Oil Sands Region (OSR) of northern Alberta, Canada, is shown in Fig 1A, and the collection locations of each species (from the cleaned data set) in the OSR are presented in Fig 1B.

**Table 1:** Summary of samples used in the analysis pre- and post-data cleaning.

Species	Pre-cleaning n	Post-cleaning n
Amphibian		
Wood Frog (Adult)	51	13
Wood Frog (Recent Metamorph)	32	8
Wood Frog (Tadpole)	44	21
Mammals		
Adult River Otter	113	113
Adult Marten	120	50
Adult Mink	23	23
Adult Muskrat	20	20
Adult Fisher	63	63
Colonial Waterbirds		
Gull Eggs	255	181
Tern Eggs	24	0
Total	694	492

**Fig 2:**
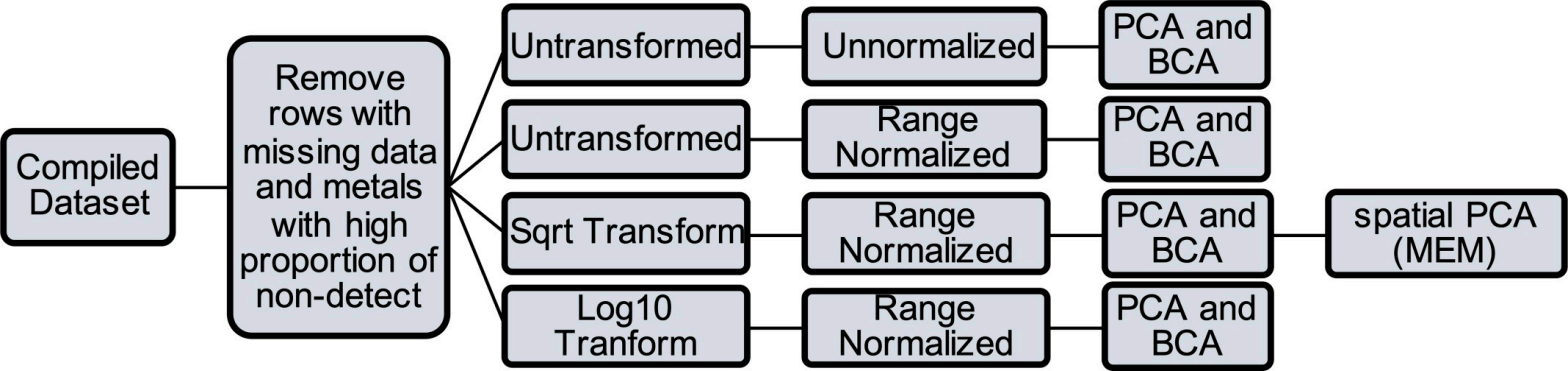
Overview of data processes and methods used in the integration and analysis of wildlife contaminants datasets. PCA = principal components analysis, BCA = between-class analysis, and MEM = Moran eigenvector map.

The dataset was assessed to ensure it met PCA assumptions, which includes a sufficient sample size and a linear correlation between covariates. Since PCA results can be influenced by outliers, it is important to transform the datasets as contaminant concentrations in ecotoxicology tend to be skewed left, with many low exposures and few high exposures. Scaling and centering the data is common in PCA algorithms [31]. We choose to do these steps manually to control and evaluate the process. First, we conducted both a log10 and square-root transformation (centering) to compare the effectiveness of the normalizing methods. Further, to integrate the datasets collected from different species and life stages, we range-normalized (scaled) by species-life stage subgroups to convert all measurements to a common scale (0–1) within each subgroup. Essentially this ranks the exposure within each subgroup [27, 32]. This is necessary to control for the differences in bioaccumulation and biomagnification rate of metals and PTEs between species and life stages and allows for contaminant concentrations to be compared and integrated [27, 33]. A workflow of the data preparation and analysis is shown in Fig 2. All analyses were completed in R 3.4.3 using the packages ade4, adespatial, spdep, and sp [34–37].

### Data analysis

We ran a between-class analysis (BCA), a subtype of PCA that assesses the variance attributed to each grouping in the dataset including site, species, and year of sample collection, before and after transformation (Fig 2) [38]. The goal of this analysis was to minimize the amount of variance explained by species differences without sacrificing the variance explained by the sample site. The square-root transformed and normalized data transformation was the optimal transformation method as that dataset retains the highest amount of total variance explained (71.2%) while minimizing the variance explained by species differences (21.3%) and maximizing the amount of variance explained by site (41.9%). The square-root transformed range-normalized data were spatially analyzed using the Moran eigenvector map (MEM) framework to assess the multivariate spatial structure of contaminant exposures in the studied wildlife. A spatially weighted matrix, which encodes the topological spatial relationships between samples, is required for the MEM. Due to the irregular distribution of the samples, a Gabriel graph with edges weighted by inverse distances was used to define the spatial connectedness [39]. The inverse distance weighting guarantees that samples closer together will have a stronger influence in the spatial PCA than samples further apart. The spatial autocorrelation of the MEM components was assessed using a Monte-Carlo Moran’s I and then mapped to quantify spatial patterns. The mapped components are interpreted using biplots/loading values.

We then used the environmental fitting (envfit: vegan package [40]) to assess the correlation between environmental variables from the Alberta Biodiversity Monitoring Institute (AMBI) including, human footprint (2015) for mining activities (mines and industrial area), active and abandoned wells, seismic lines, harvest/ cultivated lands, habitat intactness (all species) [41] and landcover from the 2010 North American Land Change Monitoring System (NALCMS) [42]. In ArcGIS 10.6 [43], a 2 km radius buffered was created around each sampling site to extract environmental variables from the local environment, which results in the percentage of land disturbed by each disturbance category and the predominant landcover class. These variables were used as the environmental predictors in the environmental fitting analysis.

## Results

**Fig 3:**
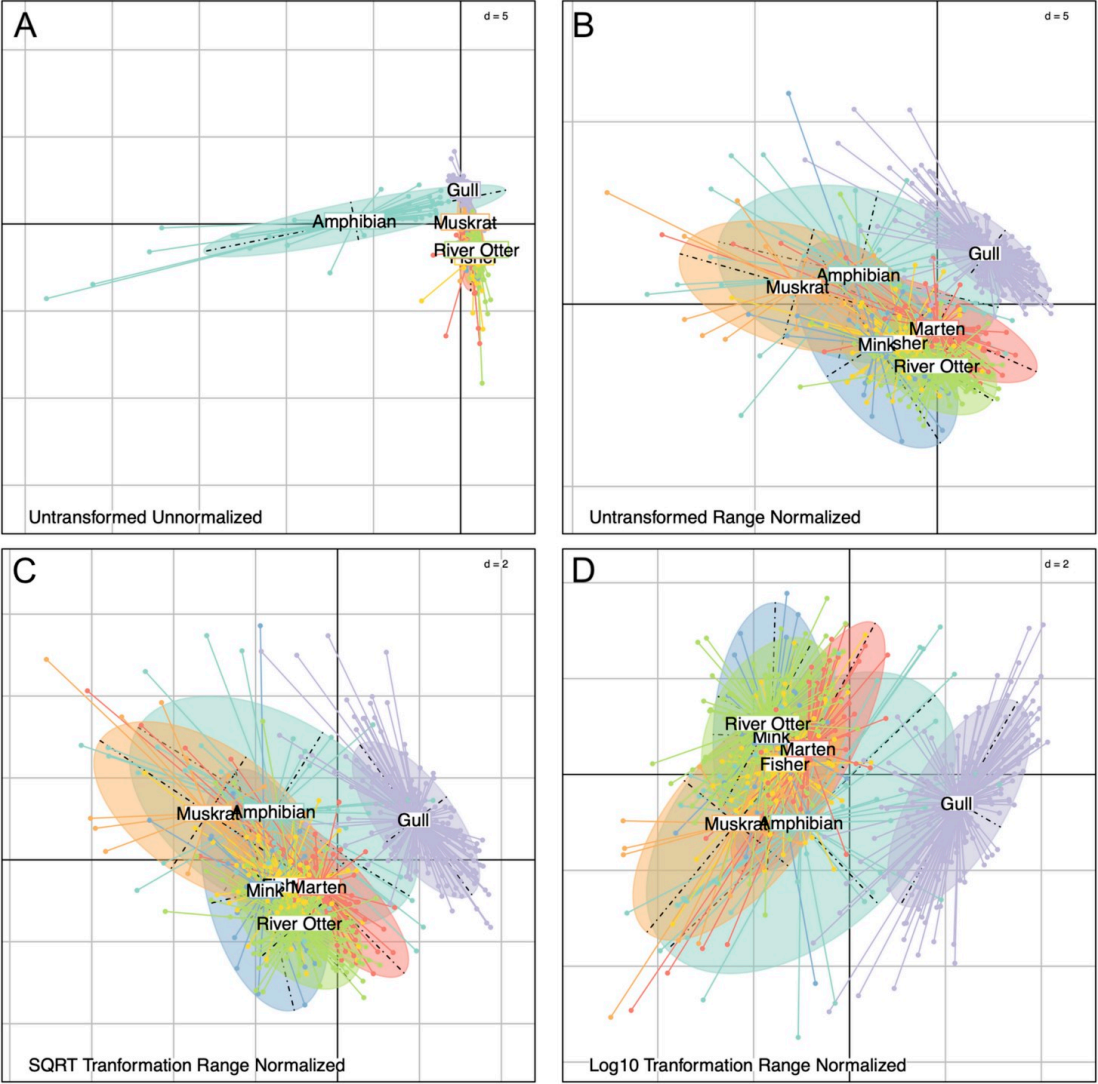
The between group variance attributed to each species group comparing the transformations. (A) untransformed and unnormalized, (B) untransformed and range normalized, (C) square-root (SQRT) transformed and range normalized, and (D) log10 transformed and range normalized. The transformation and normalization with the smallest species effect will have the most overlap between the species groups.

The BCA results comparing the two data transformation methods (square-root and log base 10) in combination with the range-normalization method are shown in Fig 3. A comparison of life stage differences is presented in S1 Fig. A lack of overlap between the amphibians and the other species in the unnormalized and untransformed data (Fig 3A) highlights the need to undertake normalization methods to make the datasets more comparable across species. After range-normalizing, the datasets were more comparable as shown by the overlap of all species in Fig 3B. The goal of the normalization is to minimize species differences, so the variance captured in the PCA will be more reflective of the site differences as opposed to the differences in the biology of different species. While the normalization method minimized the variance differences between amphibians and the other species, this also increased the variance differences between the gulls and the other species, as indicated by the separation of the gulls from the other species in Fig 3B–3D. Further, there is little difference seen between the data transformations seen in Fig 3C and 3D. The transformation has corrected the non-normal distribution of the data.

The visual patterns observed in Fig 3 were statistically tested using the Monte-Carlo simulation to quantify the amount of variance attributed to each factor (species, life stage, and site) (Table 2). Results from the Monte-Carlo simulations show that the sample site explains the most variance in the contaminant data, followed by species, and then the year the data were collected. When comparing the variance explained by each factor, transforming and normalizing the data reduces the effect of both species and life stage, compared to the untransformed or unnormalized data. After range normalization, 49.8% (p = 0.001) of the variance was explained by site in the amphibian dataset, 29.3% (p = 0.001) of the variance was explained by site in the gull dataset, and 26.4% (p = 0.001) of the variance was explained by site in the mammal dataset. The PCA and BCA results from the individual datasets (amphibians, gulls and terns, and mammals) are presented in S2 Fig.

**Table 2:** Summary from the Monte Carlo permutation test quantifying the amount of variance explained by each factor (species, life stage, and site).

	Untransformed Normalized (Raw)	Untransformed Normalized	SQRT Transformed Normalized	Log10 Transformed Normalized
Variance Explained by Site	54.9%	42.3%	43.5%	44.2%
Variance Explained by Species	35.7%	25.0%	25.6%	26.9%
Variance Explained by Year	7.1%	7.0%	7.4%	7.8%
Total	97.7%	74.3%	76.5%	78.9%

* All tests were significant at p = <0.001.

**Fig 4:**
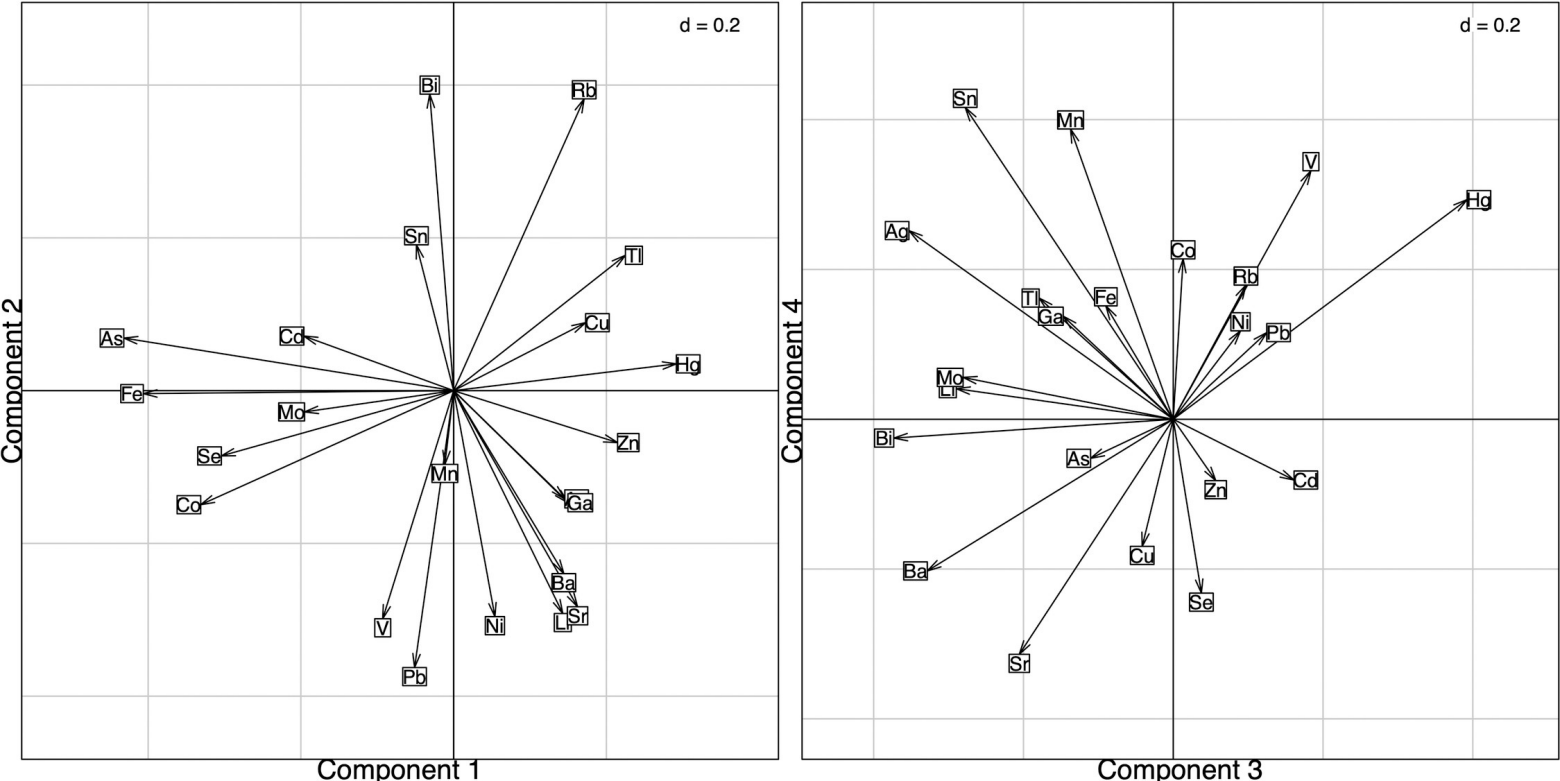
Spatial principal component analysis (sPCA) of metal and PTE concentrations in wildlife tissues using the Moran Eigenvector Maps (MEM) biplot for components 1–4. (A) Biplot between component 1 (x-axis) and 2 (y-axis) from the sPCA MEM. (B) Biplot between component 3 (x-axis) and 4 (y-axis) from the sPCA using MEM.

**Fig 5:**
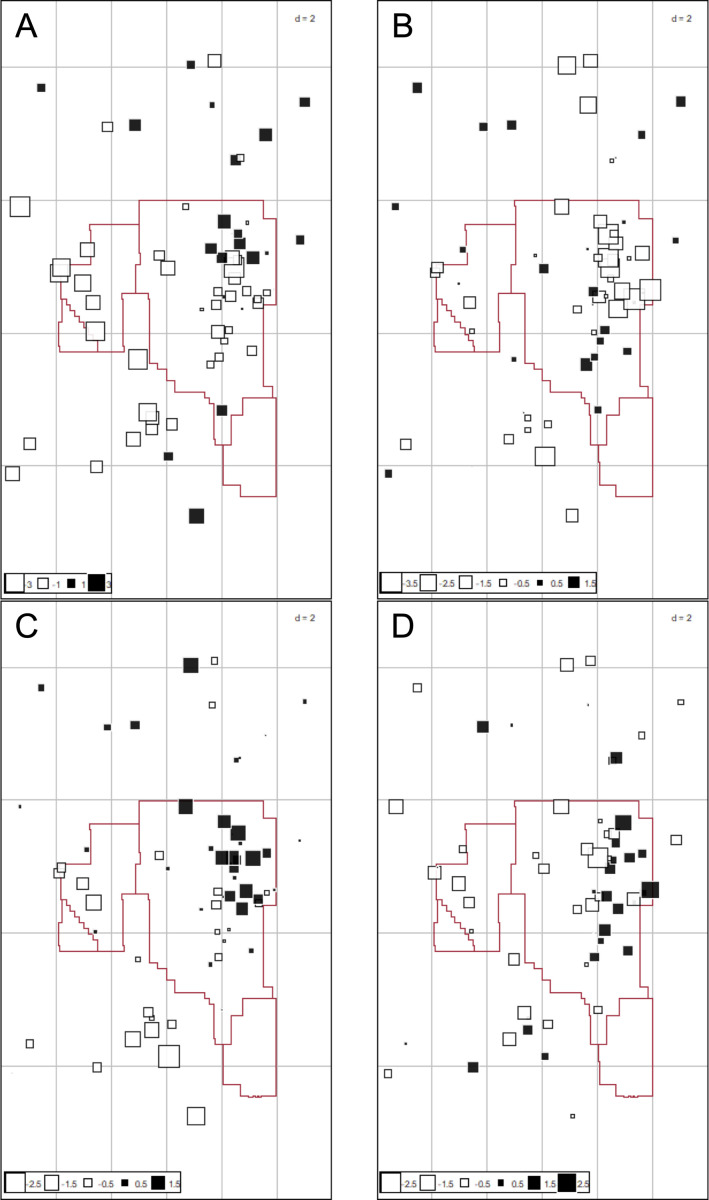
Mapped scores produced by the spatial Moran Eigenvector Maps (MEM). (A) component 1, (B) component 2, (C) component 3, and (D) component 4. These scores are interpreted using the biplots from Fig 3 and Table 2. The red box highlights the location of operating facilities in the Athabasca Oil Sands region.

The square-root transformed range-normalized data were fully interpreted using MEM to assess hotspots of complex metal exposure. The biplot from this analysis (Fig 4), and the loadings (Table 3) were used to interpret the mapped components (Fig 5).

**Table 3:** Table of loadings for Moran eigenvector maps (MEM) by site for square-root transformed and range normalized data. The metals and PTEs with the highest positive and negative loading value have been bolded.

Metal	Component 1	Component 2	Component 3	Component 4
Silver	0.15	-0.14	**-0.35**	0.25
Arsenic	**-0.43**	0.07	-0.11	-0.05
Barium	0.14	-0.24	**-0.33**	**-0.20**
Bismuth	-0.03	**0.39**	**-0.37**	-0.03
Cadmium	-0.20	0.07	**0.16**	-0.08
Cobalt	**-0.33**	-0.15	0.01	0.21
Copper	0.17	0.09	-0.04	-0.17
Iron	**-0.41**	0.00	-0.09	0.15
Gallium	0.15	-0.15	-0.15	0.14
Mercury	**0.29**	0.04	**0.39**	0.29
Lithium	0.14	-0.29	-0.29	0.04
Manganes	-0.01	-0.10	-0.14	**0.39**
Molybdenum	-0.20	-0.03	-0.28	0.05/17
Nickel	0.05	**-0.30**	0.09	0.12
Lead	-0.05	**-0.36**	0.12	0.12
Rubidium	0.17	**0.38**	0.10	0.18
Selenium	-0.30	-0.09	0.04	**-0.23**
Tin	-0.05	**0.19**	-0.28	**0.42**
Strontium	0.16	-0.28	-0.21	**-0.31**
Thallium	**0.22**	0.18	-0.18	0.16
Vanadium	-0.09	**-0.30**	**0.18**	**0.33**
Zinc	**0.21**	-0.07	0.06	-0.08

The first four components explained a total of 82.2% of the variance, which was attributed to positive spatial autocorrelation (self-similarity over space). In component 1, the positively loaded metals and PTEs were mercury, zinc, and thallium (Fig 5A). The pattern observed in the first component are similar to the location locations and loadings of the amphibian and gull PCA (Figs 1B and S2). This may be the result of the species differences not being accounted for by the normalization method (Fig 3). The negatively loaded metals and PTEs were iron, arsenic, and cobalt, which had no apparent spatial pattern (Fig 5A).

The positively loaded metals and PTEs in component 2 were rubidium and bismuth which had a dispersed pattern across the OSR (Fig 5B). The negatively loaded metals and PTEs in component 2 were lead, vanadium, nickel, and lithium. These mapped scores mostly clustered north of Fort McMurray, around the oil sands surface mining area, and the oil sands upgrading operations, as well as in the downstream receiving environment.

Component 3 was positively loaded with mercury, vanadium, and cadmium. This distinct pattern is observed around Fort McMurray, Alberta and extends northward. West of the OSR, sites are negatively loaded with barium, silver, and bismuth (Fig 5C). Component 4 was positively loaded with the metals and PTEs such as tin, manganese, and vanadium. The most negative scores are observed east of the oil sands area (Fig 5D). Negatively loaded metals and PTEs include strontium, selenium, barium, and lithium. These scores are highest in northwest Alberta and the west of the oil sands area.

**Fig 6:**
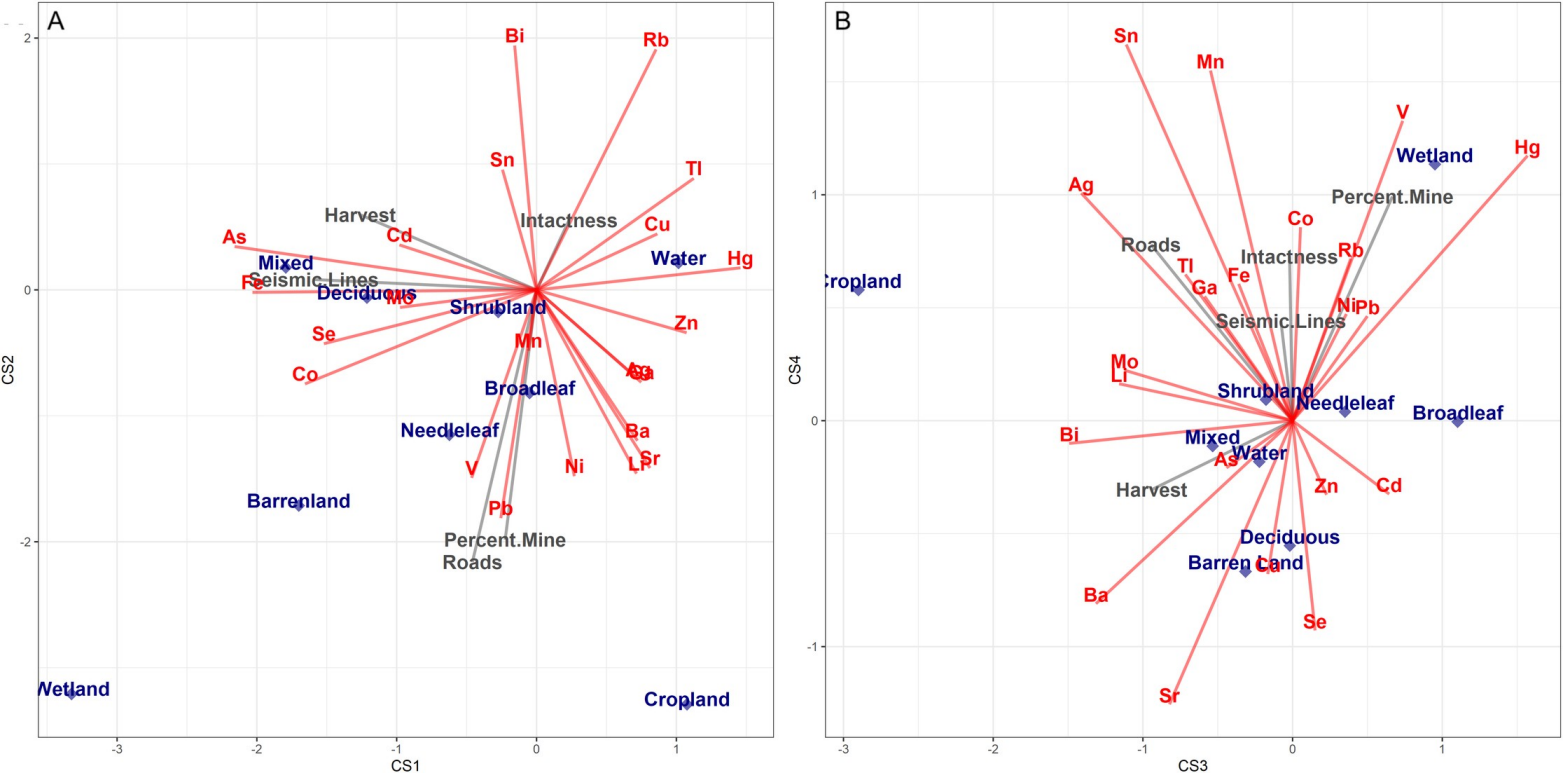
Ordination plot comparing metal and PTE concentrations in wildlife tissues with environmental variables. (A) Ordination between component 1 (x-axis) and 2 (y-axis) from the environmental fitting analysis. (B) Biplot between component 3 (x-axis) and 4 (y-axis) from the environmental fitting analysis. On both of these plots, red variables indicate metals and PTEs from sPCA, blue variables are categorical landcover environmental variables, and the grey variables are continuous environmental variables.

The environmental fitting results in Fig 6 shows the similarity in the ordination between environmental variables and metal loadings of the sPCA. In Fig 6A, mining, roads, seismic lines, and landcover are significant at (P<0.05) In Fig 6B, only landcover is significant (p<0.05). In general, mercury, lead, thallium, and vanadium have similar locations and direction on the ordination plots with variables associated with the mining environmental variable.

## Discussion

This paper demonstrates the application of methods for combining complex contaminants monitoring datasets comprised of multiple species and life stages. Range normalizing data is an important step that allows datasets to be combined and assessed for overarching spatial patterns of metal and PTE exposure, as demonstrated in Fig 3. Further, when assessing data during a spatial analysis, range normalization is superior as it retains more of the spatial structure in the data [32]. This method can also be used to help address one of the criticisms of environmental contaminants monitoring programs, that assessing a single sentinel species or single biomarker may bias a true evaluation of ecological health. However, while the range normalization is effective in making it possible to integrate the data, additional challenges remained when the sample types, locations, and chemicals analyzed did not overlap between datasets, which resulted in missing data.

Missing data is problematic for statistical methods, as it can limit analyses that can be used [44]. The simplest way of handling missing data is to delete cases with missing information or to selectively remove covariates with many missing values. We deleted cases where all metals and PTEs were not measured, which resulted in a 29% reduction in sample size and the loss of information related to contaminant exposure in terns. While this was the most straightforward method, a loss of information can lead to bias [45], which is a limitation of our model. Other methods to deal with missing data include imputation using maximum likelihood or multiple correspondence analysis. However, these methods rely on known or assumed patterns and underlying mechanisms leading to the missing data. Therefore, there must be an understanding of the underlying structure of the data for these methods to be effective [45]. Due to the complexity of this dataset and the exploratory nature of this analysis, we opted for the most straightforward method. Other methods of imputation could be explored in the future.

This paper also presents the application of a novel method for assessing the spatial patterns of multidimensional ecotoxicology data and builds on our previous work using the MEM method to assess spatial patterns of complex metal exposure in adult wood frogs in northern Alberta [27]. The results of this analysis identify key hotspots of complex metal and PTE exposure in different species and life stages in the OSR. The MEM method has successfully been used to assess spatial patterns in a variety of applications, including genetic variability [46, 47], metals in soil [48], and taxonomic and phylogenetic diversity of tadpoles [49].

The results of this research demonstrate how the traditional PCA method can be expanded to include spatial structure into the analysis of ecotoxicological data. In this analysis, component 1 identified hotspots of mercury, rubidium, and thallium exposures. The mapped positive scores are similar to the locations of gull and amphibian data from northern Alberta. Thus, this spatial pattern may be related to a species difference, rather than differences in metal and PTE body burdens between sample locations. However, these scores also exhibit a latitudinal gradient, where the scores downstream of oil sands development are larger than scores upstream. This matches patterns of mercury exposures observed in the gull and tern eggs in northern Alberta [10, 11]. Further, the spatial patterns observed in the first component also supports research demonstrating the Athabasca River as a pathway of transport of anthropogenic mercury from the OSR to downstream receiving environments [21].

Hotspots of lead, vanadium, nickel, lithium, and strontium (component 2), and mercury, vanadium, and cadmium (component 3) clustered around the open-pit mining and upgrading operations. These elements also had similar directionality on the ordination plot with an environmental variable quantifying the percentage of mining in the local environment. Local enrichment of these metals and PTEs from oil sands operations has been widely reported [5–7, 20, 50]. The common pathways of local enrichment of metals and PTEs into the environment from oil sands operations are from fugitive dust and emissions [20], and the release of metals and PTEs in an aqueous solution from process water and tailings ponds [5]. Lynam et al. [7] showed that strontium is enriched in the OSR and is a tracer for deposition of crustal dust that results from mining and land clearing activities. Further, Landis et al. [5], attributed sources of rubidium in the OSR to biomass burning and fugitive dust from mining activities. The overlap between locally enriched metals and PTEs and patterns of complex metal and PTE exposures in wildlife suggests wildlife is being exposed to a unique “fingerprint” of metals and PTEs from oil sands development.

Some components had no apparent clustering pattern. The most highly loaded metals and PTEs in these components included arsenic and iron, and a variety of other crustal elements including bismuth, strontium, rubidium, cobalt, and lithium, The mapped components that exhibit a random pattern are likely indicative of background metal and PTE exposures. In the environmental fitting ordination plot, these elements clustered with intact landscapes and deciduous and mixed forests. Northern Alberta has very complex underlying natural geological and geochemical properties. For example, monitoring data shows that northern Alberta has arsenic levels up to 10 times higher, and iron levels up to 100 times higher than background concentrations in other regions of Alberta, which is due to the naturally occurring shale deposits [51, 52]. The sPCA method discussed here helps to identify key hotspots of metal and PTE exposures as opposed to the natural background. Further, crustal elementals such as strontium may also serve as useful biomarkers for complex metal and PTE exposures that result from anthropogenic augmentation of metals and PTEs to the natural environment.

There are other sources that could contribute to the overall variance in the complex metal and PTE exposures in the biota. First, while the range normalization method attempted to address some of the issues associated with integrating contaminant data across different species and life stages, this method was not able to completely diminish the effect of species and life stage. This may be due to the bioaccumulative nature of some metals and PTEs with the age of the biota [22]. In this study, we treated all biota in the adult category similarly, but a “young” adult may have a different contaminant burden than an “old” adult. Future work should include more precise age estimations (i.e., annuli) to better control for metal bioaccumulation. Second, approximately 7–8% of the variance in the metals and PTEs could be attributed to the sampling year, which may be related to the yearly variability in production, deposition, and metal exposure in the oil sands. This variation is observed in the depositional patterns of metals and PTEs and factors affecting bioavailability, such as acidification in the OSR [6, 20, 53, 54]. Third, other environmental factors may play a role in how deposited metals and PTEs are delivered to downstream environments. Hebert [21], found that in years where the flow rate of the Athabasca River was higher, due to a greater volume snowpack melt, there were higher concentrations of Hg observed in tern eggs downstream of Oil Sands development. This highlights how the mobilization of contaminants to downstream receiving environments is mediated by complex environmental processes.

Oil Sands development is changing how wildlife uses the landscape, population densities, and species interactions both during the process of development and reclamation [4, 55]. Changes in landscape features (i.e., connectivity) and usage by wildlife can change the predator-prey relationship changing the trophic position where wildlife are feeding [55]. The trophic position has implications for altering exposures and body burdens [10, 29]. Our environmental fitting analysis showed that habitat intactness was opposite to mining and mining-associated metals and PTEs on the ordination plot (Fig 5A). This indicates that wildlife living in more intact habitats have different contaminant burdens than wildlife living in anthropogenically impacted areas. Additional research using a cumulative effects approach is needed to help better understand the complicated relationship between anthropogenic changes to landcover, wildlife usage, and contaminant burdens.

Spatial analyses of wildlife exposures can be used to identify priority metals and PTEs, determine sources, identify priority areas for further study, and help develop targeted strategies for additional monitoring or exposure reduction [13]. However, as one limitation of this method is that it only identifies statistical associations and does not allow a determination of whether the complex exposures are likely to cause adverse health effects, it is not possible to characterize the risk of the exposure. Another limitation of this paper is that we only assessed one class of contaminants in this paper. Future research should also work to incorporate other contaminant classes such as polycyclic aromatic compounds (PACs). Nevertheless, by analyzing existing monitoring data using this integrative spatial framework, we provide a cost-effective and data-led approach to identify areas that need intensive and immediate attention for monitoring and possible intervention. Based on our results, we suggest more detailed monitoring, such as a health impact assessment, is required east and downstream of oil sands development. This is of vital importance as Indigenous harvesters and other land users rely on wildlife for subsistence and food safety and security in the region. The results from this study also support an adaptive monitoring framework, where ongoing monitoring data can be incorporated into this established model, allowing ground-truthing of hotspots and identifying additional regions that may require intensive monitoring.

## Conclusion

In this paper, we demonstrate the application of an sPCA method and address current limitations of spatially assessing landscape-level complex exposures across different species and life stages. The methods we used to transform and normalize the data diminished the effect of species and life-stage differences in contaminant burdens and is a useful pre-processing step for data integration. Results show that patterns observed in the sPCA were related to patterns of environmental exposures and not a difference in species-specific accumulation rates. The spatial analysis conducted in this study demonstrates that some metals and PTEs are being anthropogenically delivered and concentrated around oil sands development. For example, levels of mercury, vanadium, lead, rubidium, lithium, strontium, and barium were elevated in multiple species around the open-pit mining area as well as regions downstream of oil sands development. The techniques presented in this paper can be applied to monitoring programs in other areas undergoing development and anthropogenic change. We also highlight areas of further research, which include the need to evaluate a broader spectrum of contaminants, including PACs, and how anthropogenic changes to the landscape result in altered contaminant burdens in wildlife.

## Supporting information

S1 Fig Principal Components Analysis (PCA) and Between Components Analysis (BCA) results. A comparison for each combination of transformation and normalization method and the effects of life stage differences.
(TIF)

S2 Fig Comparison of individual BCA results with mapped scored for amphibians, gulls, and mammals. These data have been square-root transformed and range normalized.
(TIF)
