# Supplementary figures and images for: Geospatial analysis of the patterns of chemical exposures among biota in the Canadian Oil Sands Region
Source: PLoS One. 2020 Sep 30;15(9):e0239086. doi: 10.1371/journal.pone.0239086 (PMC7526876; doi:10.1371/journal.pone.0239086)

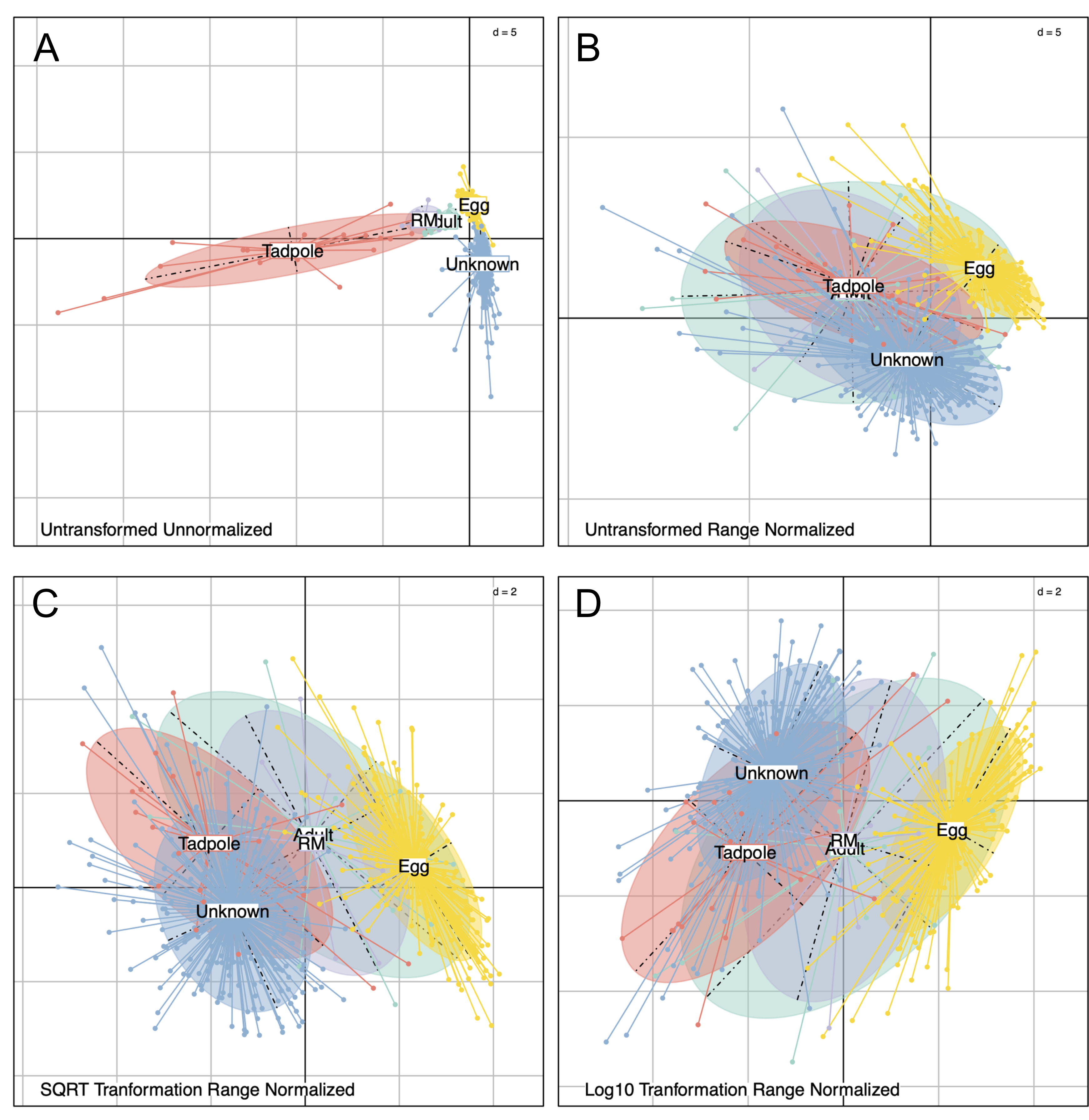

Supplement: S1 Fig — A comparison for each combination of transformation and normalization method and the effects of life stage differences. (TIF) [file pone.0239086.s001.tif]

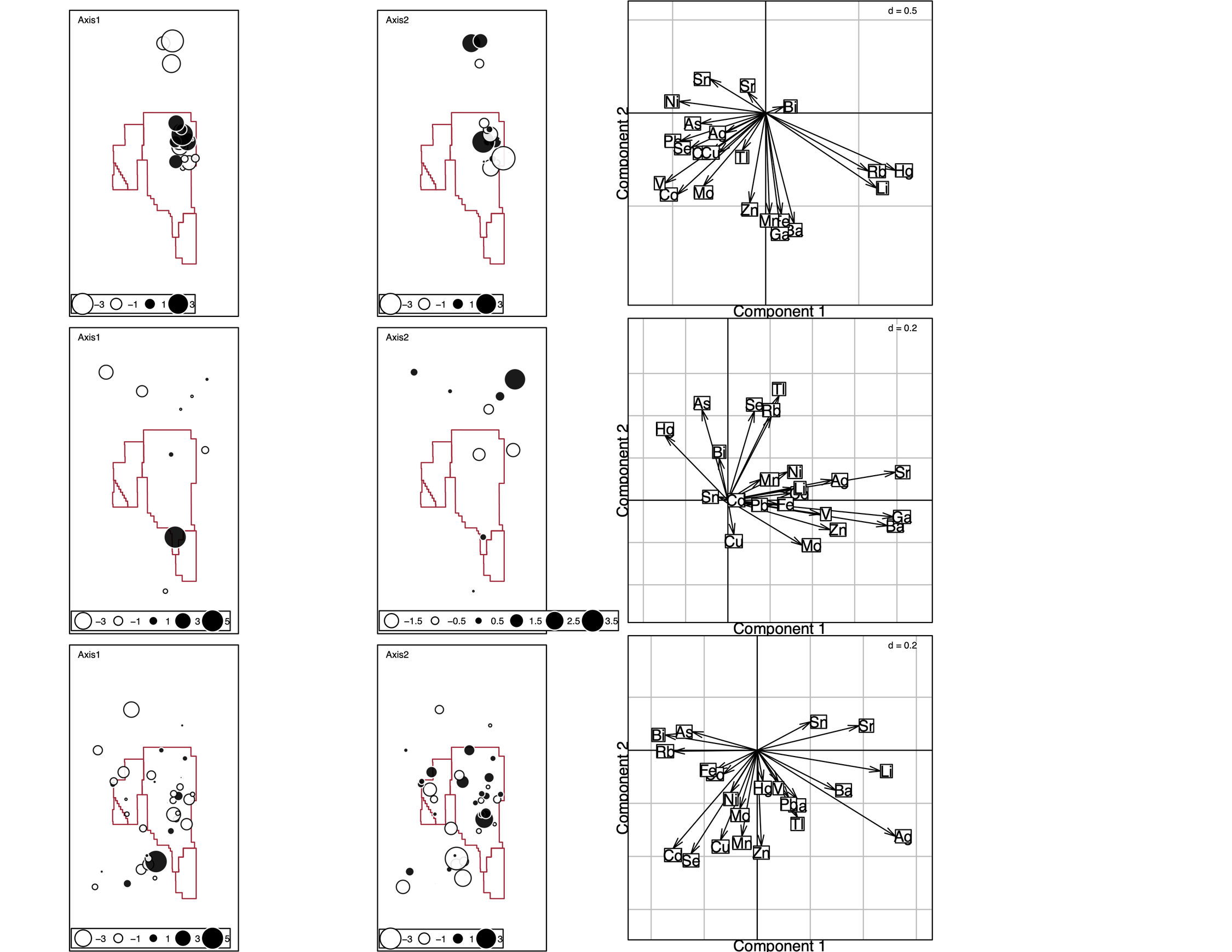

Supplement: S2 Fig — These data have been square-root transformed and range normalized. (TIF) [file pone.0239086.s002.tif]
